# Supplementary material for: Empirical methods for controlling false positives and estimating confidence in ChIP-Seq peaks
Source: BMC Bioinformatics. 2008 Dec 5;9:523. doi: 10.1186/1471-2105-9-523 (PMC2628906; doi:10.1186/1471-2105-9-523)
Supplement: Additional File 1 — A variety of html documents from the USeq web site detailing the available applications, their best usage, output file type descriptions, command line menus, etc. [file 1471-2105-9-523-S1.zip › USeqUserGuides/JavaDoc.zip!/JavaDoc/overview-frame.html]

Overview


All Classes

Packages
  
bioroot
  
bioroot.antibody
  
bioroot.oligo
  
bioroot.parsers
  
bioroot.plasmid
  
bioroot.strain
  
cgar
  
cgar.beans
  
edu.utah.seq.analysis
  
edu.utah.seq.compression
  
edu.utah.seq.data
  
edu.utah.seq.methylation454
  
edu.utah.seq.misc
  
edu.utah.seq.parsers
  
expr
  
gata.aligner
  
gata.geneGlyphs
  
gata.main
  
gata.menu
  
gata.plotter
  
igb.util
  
meme
  
selex
  
trans.anno
  
trans.bkgrnd
  
trans.cel
  
trans.graphics
  
trans.main
  
trans.misc
  
trans.qc
  
trans.roc
  
trans.tpmap
  
util.amazon
  
util.apps
  
util.apps.gump
  
util.bio.annotation
  
util.bio.calc
  
util.bio.cluster
  
util.bio.converters
  
util.bio.digest
  
util.bio.parsers
  
util.bio.parsers.gff
  
util.bio.seq
  
util.bio.wrappers
  
util.bio.xamer
  
util.gen
  
util.xml
